# Ultrasensitive detection of *Mycobacterium tuberculosis* by a rapid and specific probe-triggered one-step, simultaneous DNA hybridization and isothermal amplification combined with a lateral flow dipstick

**DOI:** 10.1038/s41598-020-73981-6

**Published:** 2020-10-12

**Authors:** Wansadaj Jaroenram, Jantana Kampeera, Narong Arunrut, Sarawut Sirithammajak, Sarinya Jaitrong, Kobporn Boonnak, Pakapreud Khumwan, Therdsak Prammananan, Angkana Chaiprasert, Wansika Kiatpathomchai

**Affiliations:** 1 Bioengineering and Sensing Technology Research Team, National Center for Genetic Engineering and Biotechnology (BIOTEC), National Science and Technology Development Agency, Pathum Thani, 12120 Thailand; 2 Tuberculosis Research Team, National Center for Genetic Engineering and Biotechnology (BIOTEC), National Science and Technology Development Agency, Pathum Thani, 12120 Thailand; 3 Department of Microbiology and Immunology, Faculty of Tropical Medicine, Mahidol University, Bangkok, 10400 Thailand; 4 Drug Resistant Tuberculosis Fund, Office for Research and Development, Faculty of Medicine Siriraj Hospital, Mahidol University, Bangkok, 10700 Thailand

**Keywords:** Infectious-disease diagnostics, PCR-based techniques

## Abstract

*Mycobacterium tuberculosis* (*Mtb*) is an insidious scourge that has afflicted millions of people worldwide. Although there are many rapid methods to detect it based on loop-mediated isothermal amplification (LAMP) and a lateral flow dipstick (LFD), this study made further improvements using a new set of primers to enhance LAMP performance and a novel DNA probe system to simplify detection and increase specificity. The new probe system eliminates the post-LAMP hybridization step typically required for LFD assays by allowing co-hybridization and amplification of target DNA in one reaction while preventing self-polymerization that could lead to false-positive results. The improved assay was named Probe-Triggered, One-Step, Simultaneous DNA Hybridization and LAMP Integrated with LFD (SH-LAMP-LFD). SH-LAMP-LFD was simpler to perform and more sensitive than previously reported LAMP-LFD and PCR methods by 100 and 1000 times, respectively. It could detect a single cell of *Mtb*. The absence of cross-reactivity with 23 non-TB bacteria, and accurate test results with all 104 blind clinical samples have highlighted its accuracy. Its robustness and portability make SH-LAMP-LFD suitable for users in both low and high resource settings.

## Introduction

Tuberculosis (TB), caused by the acid-fast bacillus *Mycobacterium tuberculosis* (*Mtb*), affects approximately one-third of the global population, and is one of the most lethal infectious diseases^1^. In 2017 alone, 10 million new TB cases were reported worldwide, killing 16% of those who had contracted it^2^. These figures reflect a tremendous burden of TB on the global economy and public health. Recently, the World Health Organization (WHO) identified several key factors as part of the strategy to put an end to TB. One is the urgent need to rapidly and accurately identify new cases in order to reduce the time-to-treatment and prevent further transmission^2,3^.

Considering the current methods for TB diagnosis, direct specimen examination using the Ziehl–Neelsen staining and polymerase chain reaction (PCR) have shown promise with their sensitivity^4–6^, but they require sophisticated equipment and complex workflows, which can pose significant challenges in low-resource settings. Since 2010, WHO has endorsed the Xpert MTB/RIF real-time PCR platform for the simultaneous diagnosis of TB and rifampicin (RIF)-resistant TB^3,4,7^. While rapid and reliable, this platform does present several practical constraints that limit its adoption for point-of-care (POC) use. These include high cost of instrumentation and the exclusive dependence on disposable cartridges^8^. Moreover, high-end instruments are technically demanding as they often necessitate regular maintenance and calibration services. These constraints can only be overcome by centralized laboratories^8,9^. Thus, simpler and more affordable diagnostic approaches are still needed, particularly in remote areas of high TB prevalence where advanced diagnostic equipment is usually unavailable^10,11^.

**Figure 1:**
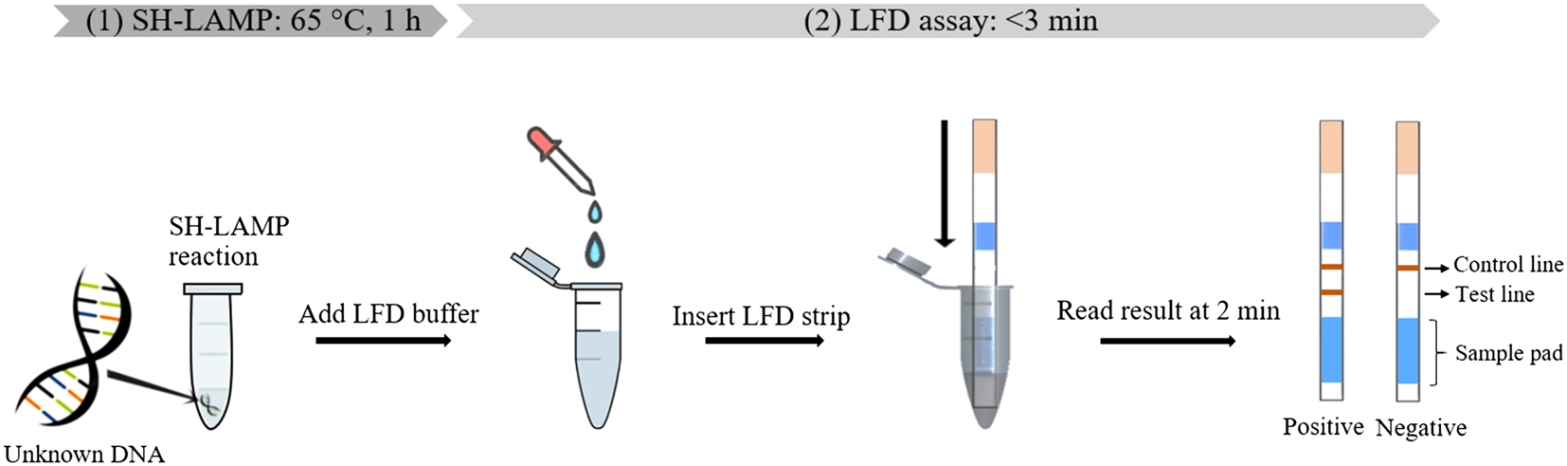
Diagram of the SH-LAMP-LFD for the detection of *Mtb.* (1) The SH-LAMP reaction was set up and incubated at 65 °C for 1 h in either a thermal cycler or a heating block for one-step simultaneous amplification of biotinylated *Mtb*-derived DNA amplicons and hybridization of the amplicons with FITC- and dSpacer-labelled probe (see Fig. 2A for the details). (2) The resulting product was diluted in 220 µL LFD buffer (1:11 ratio), followed by immersion with a generic LFD strip (Milenia Biotec, Giessen, Germany) and visualization of the test result after 2-min incubation at room temperature. In the presence of *Mtb*-DNA, the SH-LAMP amplicons complex with invisible gold-labeled anti-FITC antibodies coated on the sample pad, then travel in a buffer stream to be trapped at the test line by biotin-ligands, resulting in the appearance of a line indicative of a positive result. Non-captured gold particles move through the test line to be fixed at the red-pink, flow control line by anti-rabbit antibodies. In the absence of *Mtb* target amplicons, color appears at a control line only.

In an initial attempt to address this problem, we reported in 2013 a method to detect *Mtb*^12^ based on loop-mediated isothermal amplification (LAMP)^13^ in combination with a lateral flow dipstick (LFD) assay. The method demonstrated high specificity for *Mtb* detection in a total assay time of approximately 70 min. Although this early LAMP-LFD platform successfully addressed some existing limitations of TB diagnosis, it was open to further improvement to reduce the number of steps required to complete the assay, particularly regarding the hybridization of LAMP amplicons to the LFD probe. Here, we present a new LAMP-LFD method that is capable of detecting *Mtb* in a much simpler, more rapid and sensitive manner than previously reported. The new method is based on probe-triggered, one-step simultaneous DNA hybridization and LAMP followed by an LFD assay (i.e., the SH-LAMP-LFD method). Its working principle is illustrated in Fig. 1.

## Results

### LAMP primer design

**Figure 2:**
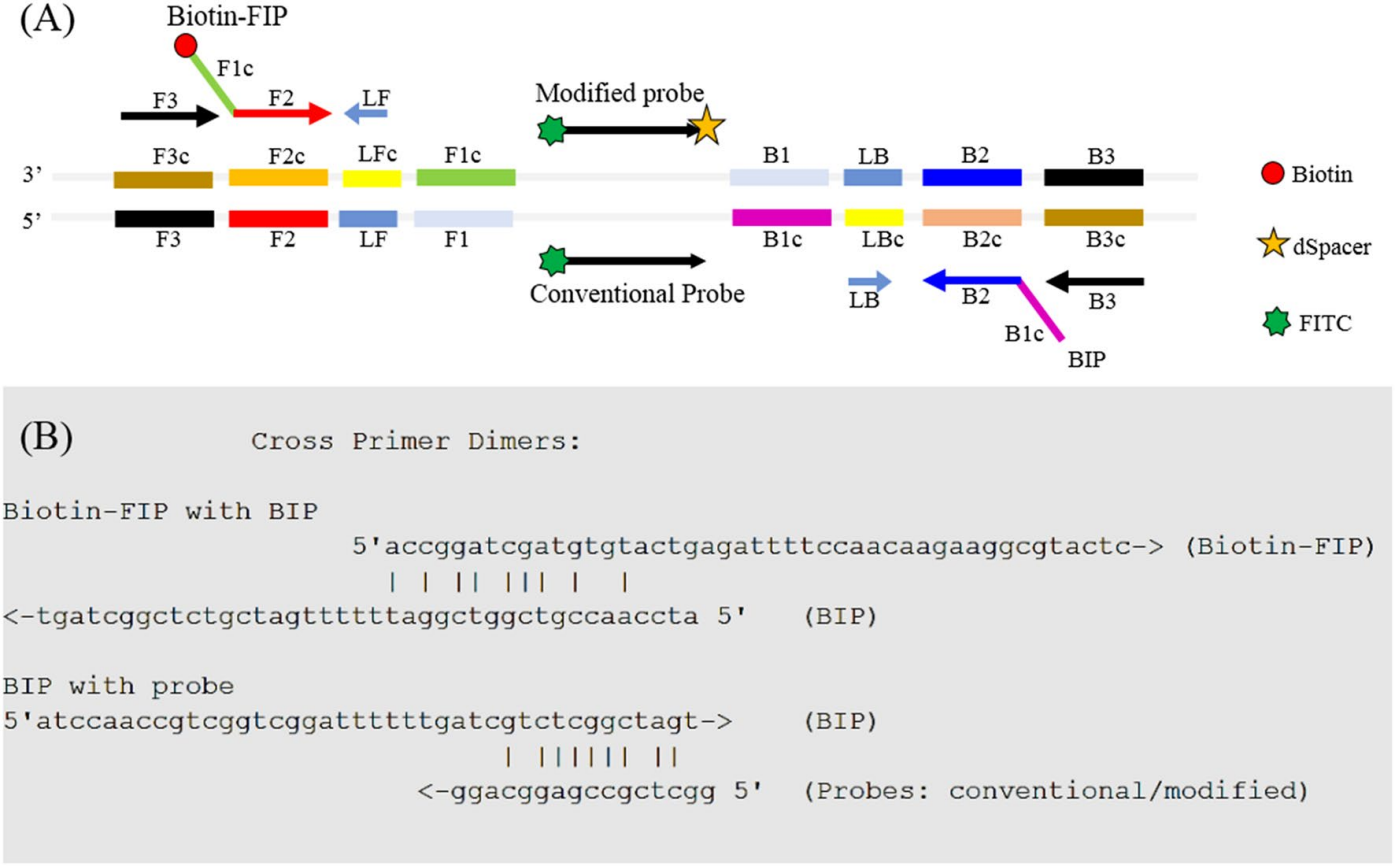
LAMP primer organization and in silico analysis. (**A**) Organization of LAMP primers [F3, B3, biotin-FIP (F1c/TTTT/F2), BIP (B1c/TTTT/B2) and LF, LB], and DNA probes (modified and conventional) on the IS6110-liked element of the *Mtb* gene (see Table 1 for additional details)*.* Arrows indicate the direction from 5′ to 3′ ends. (**B**) Primer and probe analysis by Multiple Primer Analyzer (Thermo Fisher Scientific) where the given value of the sensitivity for dimer detection was 3 (optimal, default on a scale of 1–10 where 1 is the maximum sensitive detection). Partial binding among the primers and probes were observed. Whether this phenomenon leads to self-polymerization was further evaluated.

A set of six LAMP primers (F3, B3, FIP, BIP, LF and LB) targeting the *Mtb*-IS*6110* gene sequence of *Mtb* was designed (Fig. 2A, Table 1) following the criteria listed under the Materials and Methods section. The ΔG values of all primers were less than − 4 Kcal/mol. For the LFD assay, two types of probes were used. One was conventional, and the other was newly created and called a “modified probe”. The conventional probe was labeled with FITC at 5′end, while the modified probe was tagged with FITC at the 5′ end and a dSpacer at the 3′ end. In silico analysis revealed possible cross-reactivity among primers and probes (Fig. 2B), and suggested that BIP could interact with FIP along with both types of probes. As a result, the conventional probe would most likely undergo primer–probe self-polymerization leading to false-positive signals in the LAMP-LFD, while the modified probe would not due to blocking by the dSpacer at its 3′ end (Fig. 2A).

**Table 1:** Primer and probe sequences for *Mtb*-SH-LAMP-LFD.

Name of primer/probe	IS*6110* gene position	Sequence (5′-3′)
F3	937–954	GATCGAGCAAGCCATCTG
B3	1151–1133	TGATCAGCTCGGTCTTGTA
Biotin.FIP	1037–1016/TTTT/961–979	Biotin-ACCGGATCGATGTGTACTGAGA/ TTTT/ CCAACAAGAAGGCGTACTC
BIP	1064–1081/TTTT/1125–1108	ATCCAACCGTCGGTCGGA/TTTT/ TTGATCGTCTCGGCTAGT
LF	991–1012	CCTATCCGTATGGTGGATAACG
LB	1105–1085	GTCGGAAGCTCCTATGACAAT
Modified probe	1047–1062	FITC-GGCTCGCCGAGGCAGG-C3-spacer
Conventional probe	1047–1062	FITC-GGCTCGCCGAGGCAGG

### Optimization of LAMP-LFD conditions and probe hybridization

**Figure 3:**
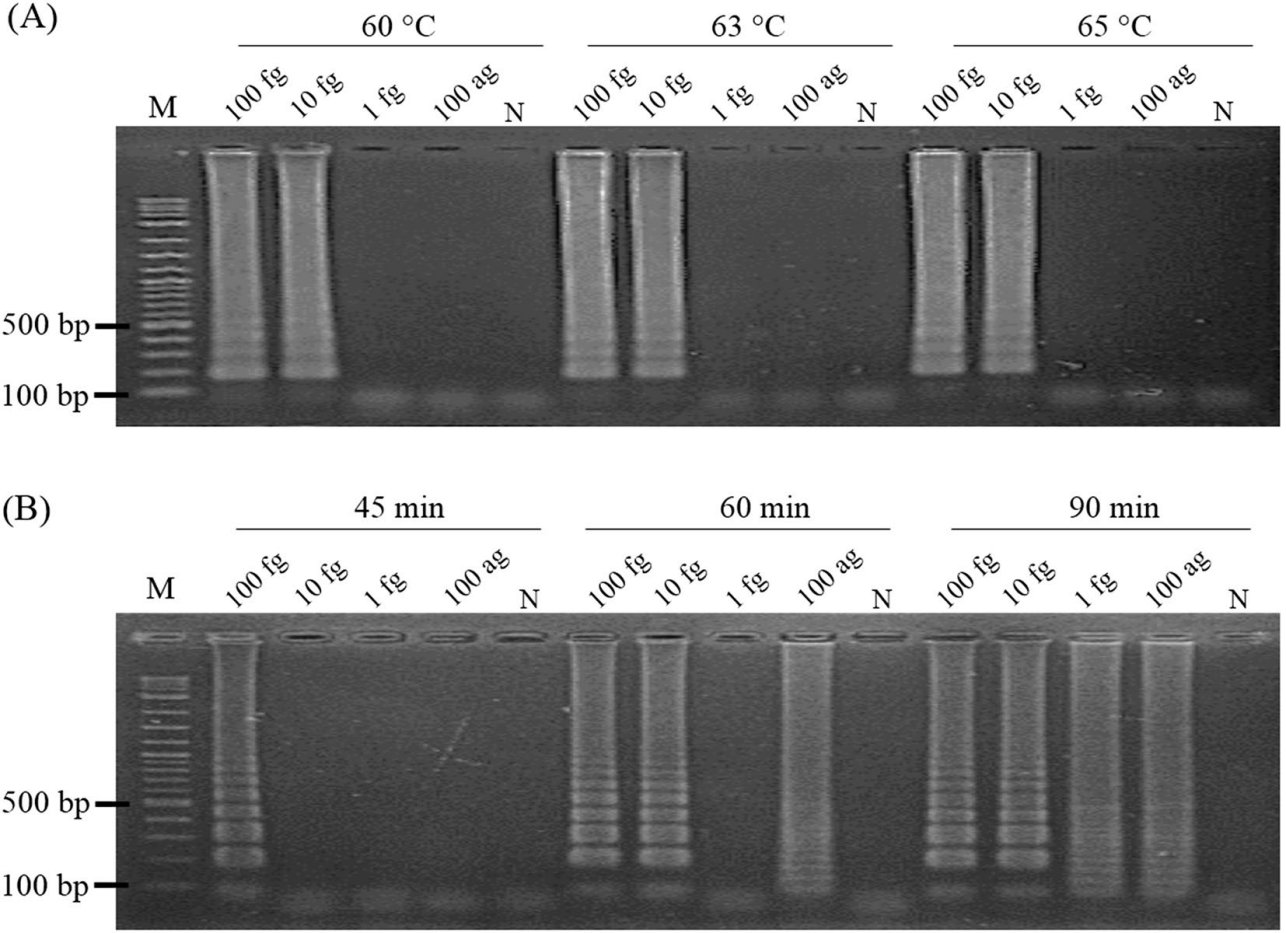
Determination of standard LAMP conditions. (**A**) Optimization of LAMP reaction temperature under 60-min incubation using tenfold serially diluted *Mtb*-DNA as a template. (**B**) Optimization of LAMP reaction time at the optimal temperature (65 °C) using varying template amounts as listed in (**A**). Amplification results using 1 fg and 100 ag templates indicate the presence of non-specific LAMP amplicons in the reactions that can be characterized by a denser ladder-liked electrophoretic pattern of DNA migration. Lanes M and N: molecular marker and negative control (DNase-free water), respectively.

**Figure 4:**
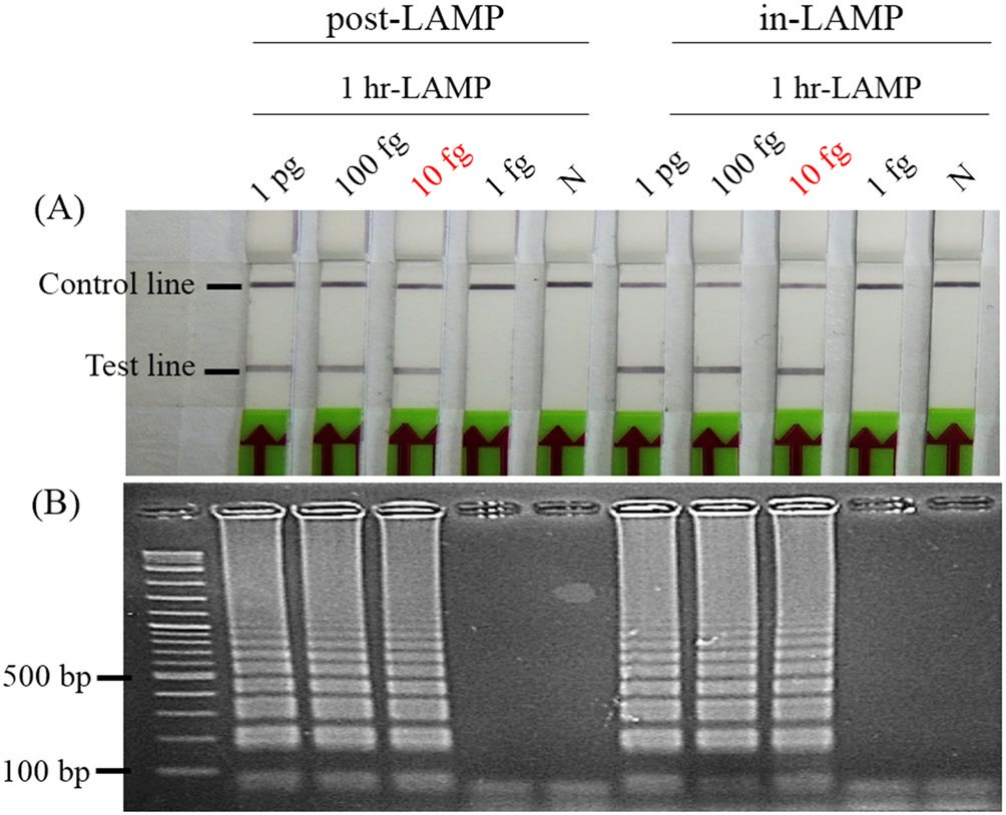
Comparative efficiency of hybridization methods. (**A**) Comparison of LFD results by different hybridization methods. In Method I (post-LAMP), LAMP products from probe-free reactions of 1 pg, 100 fg, 10 fg and 1 fg *Mtb*-DNA templates were hybridized with the conventional probe for 5 min followed by LFD the assay. In Method II (in-LAMP), LAMP reactions containing the modified probe were carried out to completion and immediately subject to LFD assay. Both hybridization methods shared identical sensitivity. N: negative control (DNase-free water). (**B**) Corresponding AGE results for the LAMP amplicons as shown in (**A**).

LAMP reactions performed at 60, 63 and 65 °C with 100 fg, 10 fg, 1 fg and 100 ag of *Mtb*-DNA template showed no difference in amplification by agarose gel electrophoresis (AGE) detection (Fig. 3A). Thus, 65 °C was selected as the standard temperature since higher temperatures generally yield higher primer specificity. For optimization of reaction time, the quantity of amplified DNA at 60 min was similar to that at 90 min (10 fg), but higher than that at 45 min (Fig. 3B). Nonspecific amplicons were observed occasionally on electrophoresis, but only at very low concentrations of the target DNA (100 ag) at 60 and 90 min. However, their presence produced no effect on the LFD readout when an appropriate probe was used. To avoid generating nonspecific amplificons that might result in false positives in subsequent experiments, 60 min was selected as the standard amplification time. To link our *Mtb*-LAMP to the LFD platform, hybridization of the DNA amplicon and probe was evaluated using conventional and modified probes. These were tested by addition to the reaction before the LAMP reaction (in-LAMP) and after it (post-LAMP) [see the “Materials and methods”, subsection Optimization of the hybridization step for the lateral flow dipstick (LFD)]. Both in- and post-LAMP methods showed comparable hybridization efficiency (Fig. 4A) with no evidence of interference with the LAMP reaction (Fig. 4B). However, the in-LAMP method was eventually chosen for subsequent experiments because it allowed for single-step visualization.

### In-LAMP hybridization is insensitive to off-target DNA

**Figure 5:**
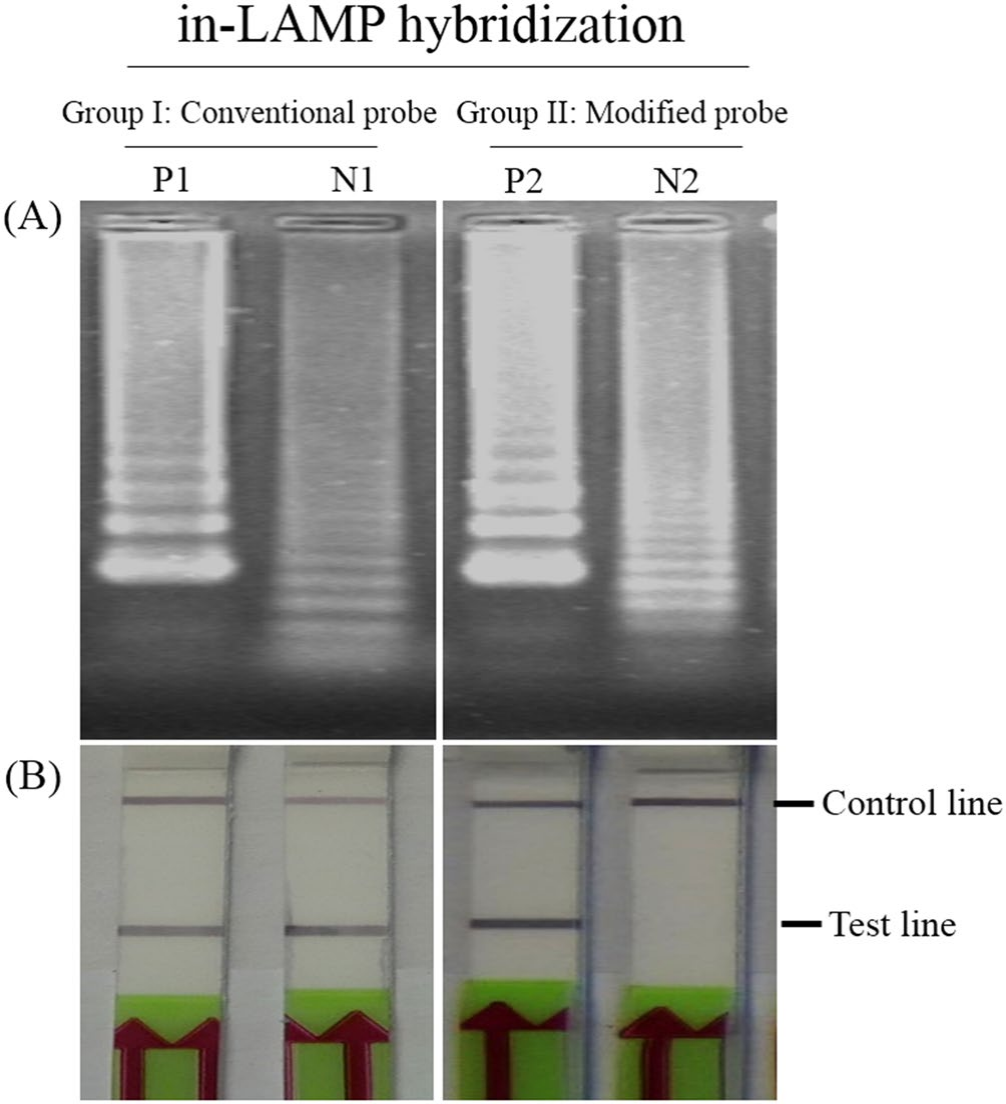
Comparative specificity of the conventional probe and the modified probe implemented on the LFD assay for detecting *Mtb*-LAMP products. (**A**) AGE results for LAMP amplicons amplified under non-optimal conditions (60 °C for 2 h) in the presence of different types of probes with and without target DNA. P1 and P2: positive control (100 ng target *Mtb*-DNA), N1 and N2: template-free reactions. (**B**) Corresponding LFD assay results of LAMP reactions from the two groups. Although non-specific LAMP amplicons are clearly observed in the AGE result (N2), use of the modified probe prevented false-positive results on the LFD.

Because of the potential for internal primer binding that might lead to self-polymerization (Fig. 2B) and of the generation of non-specific LAMP amplicons in unoptimized LAMP reactions (e.g., 1 fg and 100 ag at 90 min) (Fig. 3B), there was a valid concern regarding the possibility of cross-reactivity among the LFD probes designed to interact only with the *Mtb*-DNA sequence. To address this issue, in-LAMP hybridization products from LAMP reactions containing either a conventional probe (Group I) or a modified probe (Group II) were carried out under unoptimized conditions and compared. Both groups yielded non-specific amplicons in the absence of the target DNA (Fig. 5A, N1, N2). These were characterized by denser layers of ladder-liked DNA compared to the positive controls (Fig. 5A, P1, P2). The conventional probe (Group 1) produced a false positive signal on the LFD (Fig. 5B, N1), indicating cross-reaction with the non-specific DNA products. We postulated that this cross-reaction had occurred by self-polymerization of the biotinylated FIP with the conventional probe whose 3′end was not deactivated and was still capable of extension. This assumption was supported by the absence of such false positive signals in Group II, in which the 3′end of the modified probe was deactivated by a dSpacer (Fig. 5B, N2). Due to its lack of reactivity to off-target DNA, the modified probe was integrated into the in-LAMP hybridization method and LFD assay to establish a new detection platform herein called the SH-LAMP-LFD method.

### SH-LAMP-LFD is sensitive, specific and reliable

**Figure 6:**
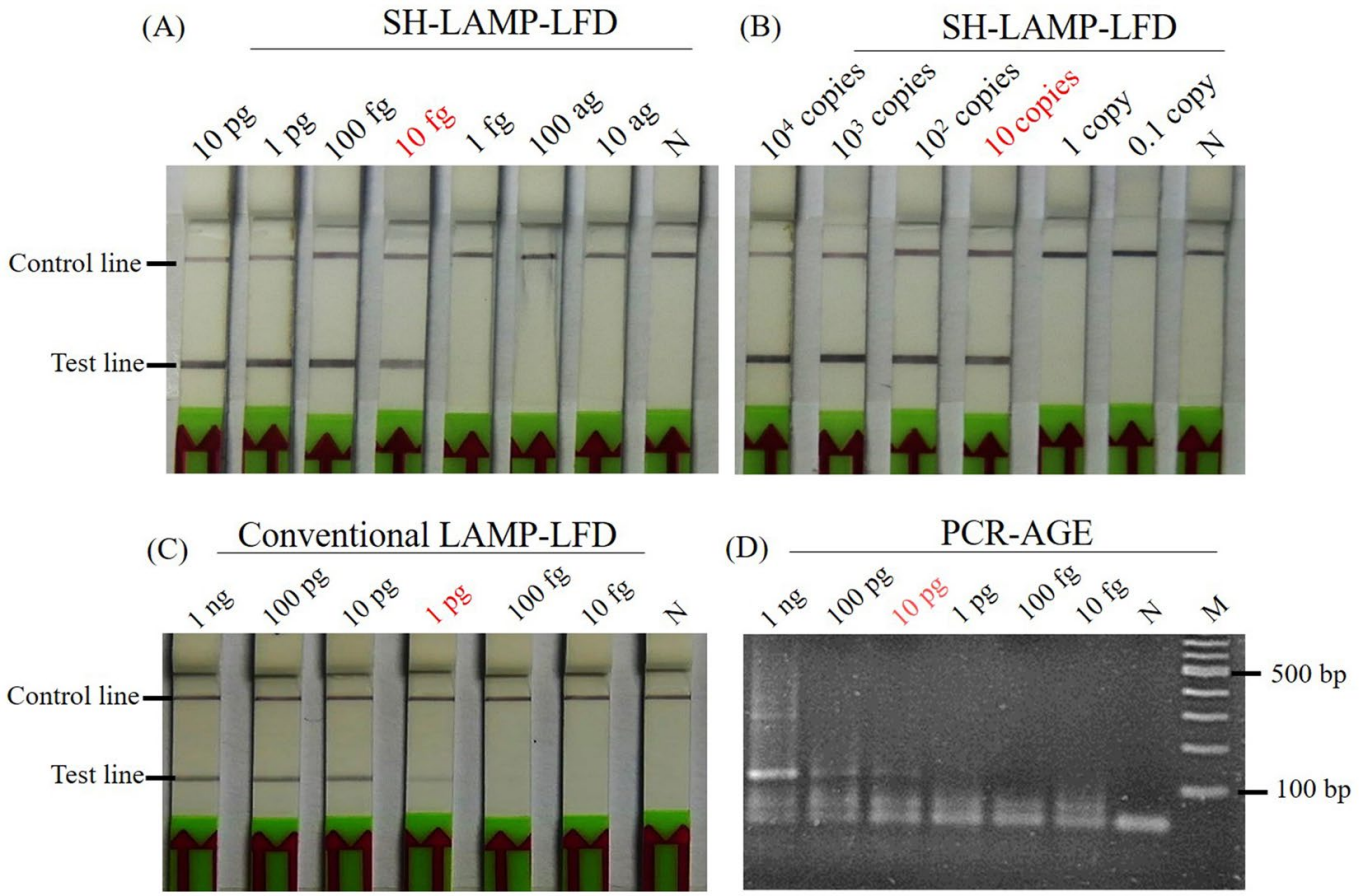
Molecular sensitivity of the SH-LAMP-LFD, conventional LAMP-LFD and PCR. (**A**) SH-LAMP-LFD results of tenfold serially diluted *Mtb*-DNA templates. (**B**) SH-LAMP-LFD results of tenfold serially diluted *Mtb*-plasmid DNA template. (**C**,**D**) LAMP-LFD results by the Kaewphinit et al. (2013) method, and PCR-AGE results by the Eisenach et al. (1991) method, respectively, using the same template set as in (**A**). The expected size of PCR the amplicon is 123 bp. Lanes N and M: negative control (DNase-free water) and molecular marker, respectively.

SH-LAMP-LFD performed with tenfold serial DNA dilutions revealed a detection limit (DL) of 10 fg *Mtb*-DNA (Fig. 6A). This was 100 times more sensitive than the conventional LAMP-LFD (Fig. 6C) and 1000 times more sensitive one-step PCR-AGE (Fig. 6D). When using recombinant plasmids as a template, SH-LAMP–LFD gave a strong positive signal with a DL of 10 copies (Fig. 6B).

**Figure 7:**
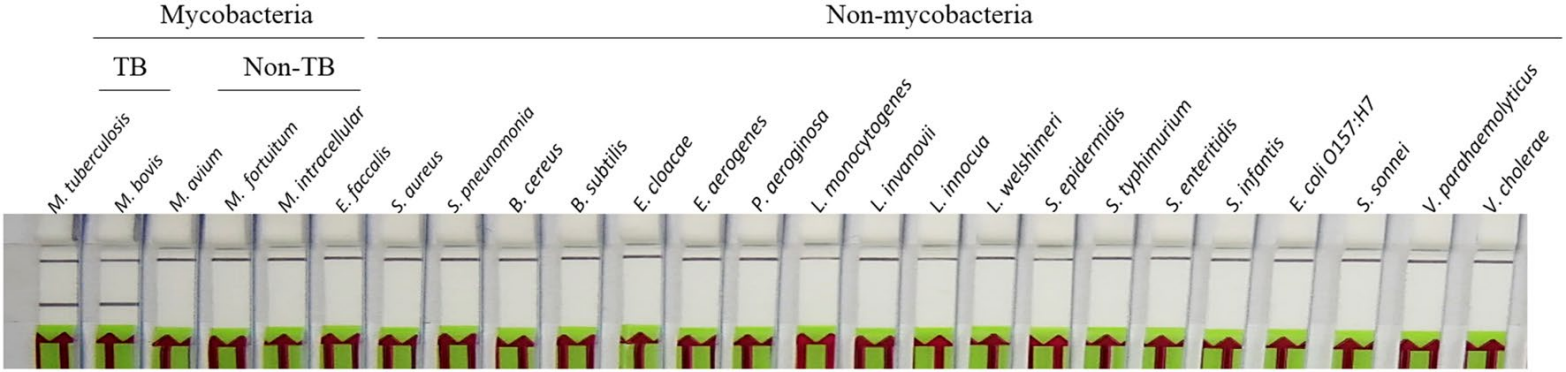
Molecular specificity of the SH-LAMP-LFD using DNA templates extracted from mycobacteria and other non-related bacteria.

To determine molecular specificity, our SH-LAMP-LFD assay was evaluated with 100 ng DNA extracted from multiple pathogens. The technique accurately detected *Mtb* and *Mycobacterium bovis* which also causes TB in humans (Fig. 7). We evaluated the diagnostic performance of our SH-LAMP-LFD with 104 sputum-derived DNA samples isolated from TB and non-TB patients, 74 test samples were identified as positive for *Mtb* while the remainders were determined as negative. Our findings were identical to the results determined by a standard culture assay (reference method) (Table 2) discussed under the Materials and Methods section. According to the reference method, the statistical sensitivity and specificity (i.e., the ability of the method to correctly identify the respective actual *Mtb*-positive, and negative samples) of our SH-LAMP-LFD were both 100%. This finding was also consistent with that of our previous study, Graphene-based electrochemical genosensor incorporated LAMP for *Mtb* detection, when the same blind samples were used^14^.

**Table 2:** Demography and *Mtb* status of samples as determined by the reference assay (standard culture), and the SH-LAMP-LFD protocol.

Source	N	Sex (M/F)	Average age (M/F)	Previous TB-Treatment (Unk/no/yes)	Anti-HIV (Unk/N/P)	Origin (North/Central/ North-east/South)	*Mtb* status	
							Positive by reference/ SH-LAMP-LFD	Negative by reference/ SH-LAMP-LFD
Chulalongkorn hospital	28	N/A	N/A	N/A	N/A	N/A	28/28	0/0
TB Laboratory, BIOTEC^a^	16	9/7	36.5/42	6/6/4	9/6/1	0/10/4/2	16/16	0/0
Siriraj hospital	30	18/12	40/44	9/15/6	17/13/0	0/19/9/2	30/30	0/0
Faculty of tropical medicine, Mahidol University^b^	30	18/12	72.2/54.8	N/A	N/A	N/A	0/0	30/30

^a^National Center for Genetic Engineering and Biotechnology.

^b^The total number of samples from this institute is 30 which are derived from 9 Influenza-infected patients: 7 for Type H3N2, and 2 for Type H1N1. The samples were prepared in various replicates in a time-and-place-independent manner prior to being given blind for the purpose of reliability testing.

N, M, F, Unk, N, P, N/A: total numbers of samples, male, female, unknown, negative, positive, not available, respectively.

These data were used to evaluate the diagnostic performance of the SH-LAMP-LFD assay (Table 3). All samples were sourced from Thailand.

## Discussion

In 2013, we reported a 3-step LAMP-LFD protocol for *Mtb* detection^12^ that consisted of DNA amplification using 4 primers (step 1), hybridization of the amplified DNA to a conventional probe (step 2), and visualization of the test result by LFD (step 3). In this current study, we improved upon our previous protocol to produce a simpler, faster, and more sensitive SH-LAMP-LFD assay (Fig. 1) for POC diagnosis of *Mtb*. The current approach employed 6 primers to enhance the performance of LAMP and a newly modified probe (Table 1 and Fig. 2) to simplify the process of detection into one reaction by merging steps 1 and 2.

For LAMP assay optimization, a reaction performed at 65 °C for 60 min was defined as optimal as it resulted in the maximum DNA amplification in the least assay time required (Fig. 3). To combine LAMP with the LFD assay, we first determined the method to efficiently hybridize LAMP amplicons with the probes. Both the in- and post-LAMP hybridization strategies showed no significant difference in LFD-positive signal generation (Fig. 4A) and no interference in DNA amplification (Fig. 4B). However, the in-LAMP method was much simpler because it could be included in the DNA amplification process. Therefore, the need for extended workflows associated with DNA-probe hybridization (i.e., multiple tube-opening and pipetting) required in the previous LFD assay setting^15,16^ was eliminated. In the reaction of in-LAMP hybridization, the probe did not self-polymerize with other primers because its DNA extension activity at the 3′-end was deactivated by dSpacer. Hence, false positive results on the LFD were precluded. In contrast, false-positives observed when using a conventional probe arose from its active 3′ -end allowing undesired extension to occur through cross-reaction with non-specific DNA (Fig. 5B, N1) and biotinylated FIP. Our observations provide supporting evidence for the self-polymerization of LAMP primers to form non-specific DNA^17,18^. Note that the generation of these non-specific amplicons was achieved under nonoptimal conditions solely for the purpose of the hybridization efficiency test. We have never observed the generation of non-specific amplicons in LAMP reactions operated under optimal conditions. When used with well-designed primers, the modified probe may prove beneficial in preventing false positives in future amplification systems that might otherwise yield non-specific DNA amplicons or primer-dimer artifacts.

Having demonstrated that the in-LAMP hybridization could accommodate the modified probe, we integrated this system with the LFD assay to establish a new platform called SH-LAMP-LFD that circumvents technical challenges associated with our previous platform. The current technique detected *Mtb*-DNA at as little as 10 fg (Fig. 6A), which was two orders of magnitude more sensitive than our previous *Mtb*-LAMP-LFD (Fig. 6C) and three more than conventional PCR-AGE (Fig. 6D). In addition, our technique achieved a DL of 10 copies when using plasmid DNA carrying the *Mtb* gene insert as a template. This level of sensitivity is theoretically equivalent to a single cell in clinical *Mtb* samples because *Mtb* typically contains multiple copies of IS*6110* sequence (up to 25 per genome)^19^, while the plasmid template was intentionally modified to harbor only one copy/molecule. This assumption, however, needs to be confirmed by determining the CFU-titer of *Mtb* in future work. Compared to the existing techniques endorsed by WHO, our SH-LAMP-LFD has a higher sensitivity than the more complex Xpert MTB/RIF platform, which was shown to detect *Mtb* down to 5 genomic copies of purified DNA/reaction or 131 CFU in *Mtb* spiked sputum^20^. It is also more sensitive than the WHO cell culture/microscopic examination method that requires as many as 10,000 cells/mL sputum.

The specificity of our SH-LAMP-LFD was demonstrated through the exclusive detection of *Mtb* and *M. bovis* (Fig. 7) against a panel of 23 other non-related pathogens. *M. bovis* is a common causative agent of TB in cattle. It is transmittable to humans through the consumption of raw milk and inhalation of aerosolized droplets from infected cattle. Although infections in humans nowadays are rare in developed countries, mainly because pasteurization effectively inactivates it in dairy products, it is still a relatively common cause of human tuberculosis in certain areas of the developing world where pasteurization is not routine^21^. Consistent with this specificity, our technique gave no false positive or false negative test results with 104 clinical samples (Table 3), making it a highly robust technique that could potentially be adopted as a specific point-of-care screening tool.

**Table 3:** Diagnostic performance of the SH-LAMP-LFD protocol.

SH-LAMP-LFD	*Mtb* status by reference assay	
	Positive	Negative
Positive	74 (TP)	0 (FP)
Negative	0 (FN)	30 (TN)
%	100 (sensitivity)	100 (specificity)
%	100 (accuracy of SH-LAMP-LFD result)	

Sensitivity = [TP/(TP + FN)]*100, specificity = [TN/(TN + FP)]*100.

Accuracy = [(TP + TN)/(TP + TN + FN + FP)]*100.

*TP* true positive, *FP* false positive, *FN* false negative, *TN* true negative.

In conclusion, despite many recent advances in TB detection technologies, there still remains a major, unmet need to develop a more practical and reliable method to make TB screening accessible to all nations. To accomplish this goal, we have developed a novel, practical and sensitive (single-cell equivalent) SH-LAMP-LFD assay that takes approximately one hour in total for the detection of TB. This technique is relatively simple to perform, yet offers100 times higher sensitivity than our previous 3-step *Mtb*-LAMP-LFD through the use of a modified probe system that enhances the detection specificity and abolishes the need for a post-LAMP hybridization step commonly needed for LAMP-LFD assays. Since this probe system has never been reported in LAMP, it serves as a model platform that could be adapted easily to detect other pathogens of interest using any applicable DNA amplification technology amendable to POC testing. It also overcomes the intrinsic drawback of visual detection of LAMP products using pH-sensitive dyes such as cresol red^22^ and xylenol orange^23,24^ that are unable to differentiate between target DNA amplicons and any off-target amplicons that might be present in the reaction mixture. This is because the dyes respond colorimetrically to a drop in pH of the LAMP solution as a result of hydrogen ion and pyrophosphate production during any DNA amplification whether it is specific or not. We believe that our method shows significant promise for rapid, preliminary TB screening that could be readily adopted in field clinics to complement conventional tests. However, in order to make it fully practical, a simple, rapid DNA extraction method should be integrated into the current workflow. Since LAMP is less sensitive than PCR to inhibition by natural components in clinical samples^18,25^, future studies should focus on investigating the feasibility of using minimally processed samples with the SH-LAMP-LFD assay.

## Materials and methods

### Sputum processing and DNA preparation

This project received approval from the Institutional Review Board (IRB*)* of the Faculty of Medicine, Siriraj Hospital, Mahidol University with Ethics Committee document No. 603/2555. The research was carried out in accordance with relevant guidelines and regulations prescribed by the above organizations. Anonymized, leftover sputum sediments from non-TB and TB patients from various collections in Thailand were used with informed consent obtained from all participants. All sputum samples were processed following the standard N-acetyl-L-cysteine–sodium citrate–NaOH (NALC-NaOH) method for digestion, decontamination and concentration^26^. The concentrated sediments were resuspended in 1 mL of 0.85% saline solution. Decontaminated sputum samples (500 µL) were washed once with Tris–EDTA (TE) buffer (pH 8.0). The supernatant from each sample was discarded and the pellet was resuspended with 500 µL TE buffer before being transferred to a 1.5-mL microcentrifuge tube containing 0.4 mL of siliconized glass beads. Cells were broken by vigorous vortexing for 10 min at room temperature followed by boiling for 20 min. Cell debris was removed by centrifugation at 12,000 × *g* for 2 min at 4 °C and the supernatant was collected. DNA was precipitated by adding 5 M NaCl and 2.5 volumes of absolute ethanol. The DNA pellet was collected, washed once with ice-chilled 70% ethanol and resuspended in 20 µL TE buffer^27^. These samples were used for evaluating the reliability of our newly developed *Mtb*-detection assay (see below).

To prepare the DNA samples for assay optimization, sputum-derived *Mtb*-DNA obtained from Siriraj Hospital, Bangkok, Thailand, was subject to quantity and quality analysis using a spectrophotometer at 260 and 280 nm. The DNA solution was serially diluted tenfold to prepare stocks containing 50 nanograms (ng)—5 attograms (ag)/µL in DNase-free water. Two microliters of the template were used in all experiments, unless otherwise stated.

### Recombinant plasmid construction

Recombinant plasmid DNA was constructed following our protocol described previously^14^. In brief, the full sequence of *Mtb*-IS*6110* gene exclusively expressed in members of the *Mycobacterium tuberculosis* complex was amplified by PCR using forward primer F (5′-GCATGTCCGGAGACTCCA-3′) and reverse primer R (5′-GTGAGTCCGGAGACTCTC-3′), yielding a 1330-bp PCR amplicon. After ligating the PCR product into plasmid pGEM-T Easy Vector (Promega, Madison, USA) according to the manufacturer’s protocol, the recombinant plasmid was transformed into 100 µL of JM109 *Escherichia coli* competent cells followed by blue-white colony selection and confirmation by PCR using the method above. Positive colonies were cultured for 16 h in 2 mL of LB broth medium containing 100 mg/mL ampicillin. The plasmid DNA was purified using Wizard Plus SV Minipreps DNA Purification System (Promega, Madison, USA), followed by spectrophotometric analysis at 260 and 280 nm. The plasmid was serially diluted tenfold to prepare stocks containing 5 × 10^7^–0.5 copies/µL. Two microliters of the template were used in the optimized LAMP–LFD reaction*.*

### LAMP primer design and optimization

LAMP primers including outer primers (F3, B3), inner primers (biotinylated FIP, BIP) and loop primers (LF, LB) were designed according to the published sequences of the *Mtb*-IS*6110* gene (GenBank accession no. X17348) using Primer Explorer version 4 (Table 1). For LFD assays, two types of probes were used. The first probe was labeled with FITC at the 5′-end and called “conventional probe”. The second probe was labeled with FITC and dSpacer at the 5′ and 3′-ends, respectively. The latter was newly explored in this study and called “modified probe”. Both were designed from the DNA sequence between F1 and B1 regions (Fig. 2A). Since our modified probe is novel, its specific design criteria have not been established. Thus, it was created based on the criteria for typical primers of PCR and LAMP primers, and LFD probes of Recombinase polymerase amplification (RPA) assays^28^ as follows: (1) Probes typically are 18–22 nt in length, (2) Palindromic and repeated sequences which could form hairpin structures within the probe sequence are avoided, and (3) Guanine and cytosine at the 3′ end are preferred as they provide more stable binding to the target sequence. All primers and probes were synthesized by Bio Basic, Canada. Further analysis revealed possible cross-reactivities among them (Fig. 2B). This led to the question as to whether or not possible cross-binding could cause self-amplification/false positive results in LAMP-AGE/LFD assays. If the answer was yes, could the method be modified to prevent it? Tests to answer these questions were carried out.

To determine the optimal conditions (temperature and time) for DNA amplification, LAMP reactions were performed following our reported LAMP protocol^14^ with minor modifications on reaction components. Briefly, the initial LAMP reaction mixture contained 2 µM each of inner primers (Biotin-labelled FIP and BIP), 0.4 µM each of loop primers (LF and LB), 0.2 µM each of outer primers (F3 and B3), 1.4 mM of dNTP mix (New England Biolabs, MA, USA), 0.6 M Betaine (Sigma-Aldrich, MO, USA), 4 mM MgSO_4_ (NEW England Biolabs, MA, USA), 8 U *Bst* 2.0 DNA polymerase (New England Biolabs, MA, USA), 1X supplied buffer, and the specified amount of template DNA in a final volume of 20 µL. DNA-free LAMP reactions were included as negative controls. For temperature optimization, LAMP reactions were carried out using a SimpliAmp Thermal Cycler (Thermo Fisher, USA) at 60, 63 and 65 °C for 1 h, followed by heat inactivation at 90 °C for 2 min. Following amplification, 5 µL of the LAMP product was visualized by 2% agarose gel electrophoresis (AGE). For the amplification time, LAMP reactions were conducted at the optimal temperature for 45, 60 and 90 min using various amounts of DNA template followed by AGE analysis. The optimized temperature and time were used in subsequent experiments.

### Optimization of the hybridization step for the lateral flow dipstick (LFD) assay

Two methods for LAMP amplicon hybridization to the DNA probe in preparation for the LFD assay were investigated and compared. Unless otherwise stated, the entire LFD assays were performed at room temperature. Method I was the commonly used, standard protocol called “post-LAMP” hybridization using a conventional probe to hybridize with LAMP products after amplification had proceeded to completion^12,15,29^. Briefly, after LAMP reactions containing 1 pg, 100 fg 10 fg and 1 fg of *Mtb* DNA and DNase-free water (negative control) were carried out at 65 °C for 60 min with no heat inactivation, they were mixed immediately with 20 pmol of conventional probe, followed by further incubation for another 5 min to allow hybridization to take place. In contrast, method II (in-LAMP) used an equal amount of the modified probe that was directly mixed into the LAMP reaction cocktail prior to amplification. After adding DNA template, the reactions were incubated at 65 °C for 60 min to allow DNA amplification and DNA-probe hybridization to occur simultaneously. Samples of the resulting products (5 µL) from each method were analyzed by AGE. To evaluate the hybridization efficiency, the remaining LAMP product (15 µL) was diluted in 165 µL (12 fold) with the LFD buffer (1X phosphate buffered saline with 0.1% Tween 20). Then an LFD stick (Milenia Biotec, Giessen, Germany) was immersed in the diluted solute for 2 min prior to observing the test outcome. The results were compared with AGE. The optimal hybridization method was selected for all subsequent LFD experiments. Note that in tests where LAMP products were not taken out for AGE analysis, the total amount of LAMP product in a 20-µL reaction volume was directly diluted in 220 µL buffer (same ratio as above).

### Sensitivity of the in-LAMP hybridization to off-target DNA

The ability of the conventional and modified probes for in-LAMP hybridization to discriminate *Mtb*-LAMP products from non-related DNA was evaluated. LAMP reaction premix without target DNA was prepared and divided into 2 groups: Group (I) was supplied with 20 pmol of the conventional probe, and Group (II) was supplied with the modified probe of the same amount. Both groups were incubated under non-optimized conditions (60 °C for 2 h) to allow for the production of non-specific amplicons. A reaction with 100 ng *Mtb*-DNA was included as a positive control for each group. The products were analyzed by AGE and LFD assay for comparison. The probe exhibiting no cross-reactivity with off-target DNA was chosen for the in-LAMP hybridization approach. To avoid confusion, the combined technique will be mentioned in this manuscript as the “SH-LAMP-LFD” which is short for the “probe-triggered one step, simultaneous DNA hybridization and LAMP combined with LFD” method.

### Comparative sensitivity of SH-LAMP-LFD, LAMP-LFD and one-step PCR-AGE

Tenfold serial dilutions of *Mtb*-DNA (1 ng, 100 pg, 10 pg, 1 pg, 100 fg, 10 fg, 1 fg, 100 ag and 10 ag) and plasmid DNA (10^4^, 10^3^, 10^2^, 10, 1 and 0.1 copies) were tested by the SH-LAMP-LFD method. The same set of DNA templates (plasmid excluded) was further tested by the other two standard protocols: commonly accepted LAMP-LFD^12^, and one-step PCR^30^.

### Specificity of the SH-LAMP–LFD

The specificity of the SH-LAMP-LFD was examined using 100 ng template DNA prepared from each of 5 mycobacterium species (*Mycobacterium tuberculosis*, *M. bovis*, *M. avium*, *M. fortuitum* and *M. intracellulare*), and 20 other non-related bacteria of significance as pathogens or possible contaminants in samples (*Enterococcus faecalis*, *Staphylococcus aureus*, *S. pneunomonia*, *Bacillus cereu BCC 6386*, *B. subtilis BCC 6327*, *Enterobacter cloacae*, *Enterobacter aerogenes DMST 1333*, *Pseudomonas aeroginosa*, *Listeria monocytogenes ATCC 19,115*, *L. ivanovii ATCC 700,402*, *L.innocua DMST 9011*, *L. welshimeri DMST 20,559*, *Salmonella epidermidis TISTR 518*, *S. typhimurium ATCC 13,311*, *S. enteritidis ATCC 13,076*, *S. infantis DMST 26,426*, *Escherichia coli O157:H7 ATCC 35,150*, *Shigella sonnei*, *Vibrio parahaemolyticus ATCC 17,802* and *V. cholerae O1 DMST 22,115.* All mycobacterium samples were obtained from the Faculty of Tropical Medicine, Mahidol University (FTMMU), Thailand. Those labelled with DMST, ATCC and BCC were purchased from the Department of Medical Sciences (DMST, Thailand), the American Type Culture Collection (ATCC, Manassas, Va) and BIOTEC Culture Collection (BCC, Pathum Thani, Thailand), respectively. The remaining samples were obtained from various sources including the Department of Biology, Faculty of Science and Technology, Rajamangala University of Technology, Pathum Thani, Thailand (*E. faecalis*), Thailand Institute of Scientific and Technology Research (*S. epidermidis TISTR 518*), and Thailand Bioresource Research Center (*S. aureus*).

### Reliability of the SH-LAMP–LFD for clinical samples

The SH-LAMP-LFD was evaluated for its reliability to detect 104 DNA samples extracted from sputum sediments of patients of known TB status as determined by WHO’s standard culture assay, reference method^31^. Briefly, the left-over of tested samples obtained from the routine diagnostic method using cultivation on Loewenstein Jensen medium and identified as *M. tuberculosis* positive by an in-house one-tube nested PCR method served as a gold standard^26,32^. The samples were anonymized and provided by Chulalongkorn Hospital, National Center for Genetic Engineering and Biotechnology (BIOTEC), Siriraj Hospital and the Faculty of Tropical Medicine, Mahidol University. All test samples were blinded prior to LAMP analysis to prevent diagnostic bias during result determination. The test results and sample demographics (Table 2) were used to evaluate the diagnostic performance of the SH-LAMP-LFD (Table 3).

### Ethics approval

This project received approval from the Institutional Review Board (IRB*)* of the Faculty of Medicine, Siriraj Hospital, Mahidol University with Ethics Committee document No. 603/2555.
